# Vitamin D status of tuberculosis patients with diabetes mellitus in different economic areas and associated factors in China

**DOI:** 10.1371/journal.pone.0206372

**Published:** 2018-11-01

**Authors:** Xin Zhao, Yanli Yuan, Yan Lin, Tiejuan Zhang, Yunlong Bai, Demei Kang, Xianhui Li, Wanli Kang, Riitta A. Dlodlo, Anthony D. Harries

**Affiliations:** 1 Beijing Hospital, National Center of Gerontology, Beijing, China; 2 Jilin Provincial Academy of Tuberculosis Control and Prevention, Changchun, China; 3 International Union Against Tuberculosis and Lung Diseases, Paris, France; 4 International Union Against Tuberculosis and Lung Disease, Beijing, China; 5 Liaoyuan City Tuberculosis Institute, Liaoyuan, China; 6 Jilin City Tuberculosis Institute, Jilin, China; 7 Beijing Chest Hospital, Capital Medical University, Beijing, China; 8 Beijing Tuberculosis and Thoracic Tumor Research Institute, Beijing, China; 9 London School of Hygiene and Tropical Medicine, London, United Kingdom; Mahidol University, THAILAND

## Abstract

**Background:**

Vitamin D could be a mediator in the association between tuberculosis (TB) and diabetes mellitus (DM). A large scale multi-center study confirmed that TB patients with DM had significantly lower serum vitamin D level compared with those without DM and reported that DM was a strong independent risk factor for vitamin D deficiency.

**Objectives:**

This study was undertaken to determine amongst patients with both TB and DM living in different economically defined areas in China: i) their baseline characteristics, ii) their vitamin D status and iii) whether certain baseline characteristics were associated with vitamin D deficiency.

**Methods:**

In DM-TB patients consecutively attending seven clinics or hospitals, we measured 25 hydroxycholecalciferol at the time of registration using electrochemiluminescence in a COBASE 601 Roche analyser by chemiluminescence immunoassay. Data analysis was performed using chi square test and multivariate logistic regression.

**Results:**

There were 178 DM-TB patients that included 50 from economically well-developed areas, 103 from better-off areas and 25 from a poverty area. Median vitamin D levels in well-developed, better-off and poverty areas were 11.5ng/ml, 12.2ng/ml and 11.5ng/ml respectively. Amongst all patients, 149 (84%) had vitamin D deficiency—91 (51%) with vitamin D deficiency (10–19.9 ng/ml) and 58 (33%) with severe deficiency (< 10 ng/ml). There was a significantly higher proportion with vitamin D deficiency in the poverty area. The adjusted odds of vitamin D deficiency (25-(OH)D_3_ <20 ng/ml) were significantly higher in those with longer history of DM (P = 0.038) and with HbA1c≥10% (P = 0.003).

**Conclusion:**

Over 80% of TB patients with DM in China were vitamin D deficient, with risk factors being residence in a poverty area, a long duration of DM and uncontrolled DM. TB programme managers and clinicians need to pay more attention to the vitamin D status of their patients.

## Introduction

Diabetes mellitus (DM) is a chronic condition that occurs when the body cannot produce or effectively utilize insulin, which results in increased levels of glucose in the blood stream (hyperglycaemia) causing metabolic disorders of many organs over time [1]. During the past few decades, socio-economic development, urbanization and life style changes have led to most countries undergoing a significant epidemiological transition, and this has resulted in a rapidly increasing burden of DM. In 2015, there were an estimated 415 million people living with DM globally, with numbers set to rise to nearly 650 million by 2040 [1]. DM is associated with several co-morbidities, one of which is a three times higher risk of developing tuberculosis (TB) compared with the general population [2,3]. In 2012, the population attributable fraction of DM for adult TB cases globally was estimated at 15% with the number of adult TB cases associated with DM being 1,042,000 [4].

Vitamin D is a secosteroid which is both synthesized in the skin by the action of sunlight and ingested in the diet. The vitamin is metabolized first in the liver to 25-hydroxycholecalciferol [25-(OH)D_3_] and then in the kidney to 1,25- dihydroxycholecalciferol [1,25-(OH)_2_D_3_], an immunologically active hormone [5]. In the context of DM and TB, vitamin D may have an important role. Experimental research has found that vitamin D and its metabolites regulate the function of pancreatic ß-cells and influence insulin secretion [6]. Vitamin D also plays a key role in human innate and adaptive immunity [5], and assists mononuclear phagocytes to suppress the intracellular growth of *Mycobacterium tuberculosis* (*MTB*) after initial infection [7,8]. Recent research has found that persons with type 2 DM with low serum vitamin D levels have impaired monocyte function and therefore reduced capacity to restrict the intracellular growth of *MTB*, and this may be one of the factors linking DM to an increased risk of TB [9].

For several decades, there has been evidence to show that people with vitamin D deficiency have a significantly higher risk of developing active TB compared with those who have normal vitamin D levels [7,8,10]. In turn, TB patients in general have also been reported to have a higher likelihood of vitamin D deficiency compared with normal controls [11,12]. A small scale study in urban China, using liquid chromatography-tandem mass spectrometry, reported significant differences in vitamin D levels between patients with TB, patients with TB and pre-DM and patients with TB and DM [13]. A larger scale multi-center study in both urban and rural China, using the electrochemiluminescence (ECLIA) method, confirmed these findings and reported that DM was a strong independent risk factor for vitamin D deficiency [14]. However, there have been no large scale studies looking at associations between vitamin D levels and DM related risk factors in TB patients who have DM in routine programmatic settings. Such information would be useful to determine who particularly might be at risk of developing vitamin D deficiency so that this can be prevented or better identified in the future.

We therefore conducted a study in patients with both DM and TB and aimed to determine in relation to their economically-defined residence: i) their baseline characteristics, ii) their vitamin D status and iii) whether certain baseline characteristics were associated with vitamin D deficiency.

## Material and methods

### Design and settings

This was a multi-centre cross-sectional study carried out in seven TB clinics and hospitals within the routine health services in Jilin Province and Beijing, China.

Seven TB clinics and hospitals in both urban and rural areas were selected for this study. The selection of the clinics and hospitals was based on the broadly defined economic development levels of the catchment areas, a sufficient proportion of TB patients with DM, the availability of essential laboratory facilities and the willingness of the staff to participate in the study without requirements of additional funding support. We selected Beijing to represent an economically well-developed area; Liaoyuan City and Tonghua City as economically better-off urban areas; Dongfeng County, Meihekou County and Tonghua County as economically better-off rural areas; and Daan County as a poverty stricken area.

### Diagnosis of TB

All TB patients were diagnosed, registered, and managed according to the guidelines of the China National TB Control Program; which is in accordance with those recommended by the World Health Organization (WHO) [15,16].

### Diagnosis of DM

The diagnosis of DM was made in accordance with the WHO guidelines [17]. Patients were diagnosed with DM either as a result of already being diagnosed by a registered medical institution and documented in the clinic records, or as a result of a fasting blood glucose (FBG) ≥ 7.0 mmol/L being identified at the time of TB registration or at the time of TB diagnosis from another health facility regardless if they were on treatment for hyperglycaemia.

### Patient recruitment

TB patients with DM who were included in this study were those either presenting to TB services by themselves with symptoms suggestive of TB or having been referred from other facilities in the same or nearby catchment area. The patients included were ≥18 years and newly registered with any type and category of TB but also with DM according to national and international guidelines. In order to have accurate data on serum vitamin D levels, we excluded the following persons from the study: a) being positive for human immunodeficiency virus (HIV), b) pregnant or lactating women, c) having Aspartate aminotransferase (AST) or Alanine aminotransferase (ALT) ≥3 times the upper limit of normal level as hepatic dysfunction may alter vitamin D metabolism, d) receiving vitamin D or Vitamin D analogues for any reason, e) receiving corticosteroid treatment for any reason, and f) receiving anti-cancer therapy as many drugs disturb hepatic function which alter vitamin D metabolism.

### Fasting blood glucose (FBG) and glycocylated haemoglobin (HbA1c) measurement

Venous blood samples were collected for FBG after a minimum of 10 hours overnight fast before anti-TB treatment commenced. FBG measurements were done in accordance with national guidelines which stipulate that a FBG is carried out using a venous plasma and biochemical analyzer with cut-off thresholds based on those recommended by the WHO [17]. Venous blood samples were collected for HbA1c as above, and HbA1c was analysed using the cation exchange method with a high performance liquid chromatography performed by G8-TOSOH, Japan [18].

### Blood sample collection and vitamin D measurement

Five ml venous blood sample was taken immediately after their TB registration and before anti-TB treatment was started. All blood samples were taken following an overnight fast of at least 10 hours. The samples were centrifuged within 2 hours to separate the serum and stored at -70^。^C until vitamin D analysis was undertaken.

25-hydroxycholecalciferol [25-(OH)D_3_], was measured in the Beijing Hospital laboratory with the electrochemiluminescence (ECLIA) method that determines serum 25-(OH)D_3_ levels in a COBASE 601 Roche analyser using a chemiluminescence immunoassay (CLIA). Reagents were supplied by Roche (Switzerland) with a normal measurement range of 3–70 ng/ml. Vitamin D levels were classified according to the standard definitions of vitamin D status: 25-(OH)D_3_ ≥ 30 ng/ml = normal; 25-(OH)D_3_ between 20–29.9 ng/ml = insufficient vitamin D; 25-(OH)D_3_ between 10–19.9 ng/ml = vitamin D deficiency; 25-(OH)D_3_ between 0–9.9 ng/ml = severe vitamin D deficiency.^8^

### Data collection and analysis

Individual patient data on demographic characteristics, TB symptoms, TB treatment history, complications, cigarette smoking status and DM related characteristics were collected. The data were entered to a MS Office Excel (Microsoft, Redmond, WA, USA) datasheet without patient names and other personal identifiers by the principal investigator and analysed using SPSS software for Windows, version 13 (SPSS Inc., Chicago, IL, USA). Shapiro-Wilk Test was used to examine normality distribution of vitamin D values. Nonparametric tests were used to compare median levels of vitamin D [25-(OH)D_3_] between groups. Categorical comparisons of various 25-(OH)D_3_ levels between DM-TB patients living in different economic areas were carried out using the chi square test. All P values are 2-tailed. Relationships between 25-(OH)D_3_ and baseline exposure variables were evaluated with odds ratios (OR) and 95% confidence intervals using logistic regression. Levels of significance were set at 5%. Variables with unadjusted ORs for which the *P* value was <0.05 were included in a multivariate logistic regression model.

### Ethics approval

The research proposal was approved by the health authorities in the implementing sites. The Ethics Advisory Group, International Union Against Tuberculosis and Lung Disease, Paris, France, formally approved this study (EAG number: 102/15). Patients recruited to this study were informed and agreed to participate. The Union model of Informed Consent Forms was used and signed by the patients.

## Results

There were 178 TB patients with DM consecutively registered in this study. They were 129 males (72%) and 49 females (28%). Of these, 50 were from the economically well-developed area, 103 from the economically better-off urban and rural areas and 25 from the poverty area. The mean age was 56 years (Range 25–89). Patients comprised 73 with smear-positive pulmonary TB, 92 with smear-negative pulmonary TB and 13 with extra pulmonary TB (EPTB).

Baseline characteristics in relation to the type of economical area that the patients resided in are shown in **Table 1**. Between the different economically affected areas, there were no significant differences in patient characteristics with regards to age, gender, category of TB (new and previously treated), DM history and level of HbA1c. However, there were some significant differences in relation to urban / rural residency and type of TB. To further understand the significant differences among the three economic areas, we performed a post-test and the results are shown in **Table 2** and **Table 3**. There were higher proportions of patients with urban residence and confirmed smear-positive pulmonary TB in the well-developed areas compared with better-off areas and the poverty area. However, there were no significant differences between patients from better-off areas and the poverty area.

**Table 1:** Baseline characteristics of patient s with tuberculosis (TB) and diabetes mellitus (DM) in China.

Characteristics		Number (%) of the patients residing in different economic areas			*χ*^2^	*P* value
		Well developed (N = 50)	Better-off (N = 103)	Poverty (N = 25)		
Gender	Male	40(31.0)	73(56.6)	16(12.4)	2.452	0.294
	Female	10(20.4)	30(61.2)	9(18.4)		
Age	<50	17(29.3)	33(56.9)	8(13.8)	0.063	0.969
	≥50	33(27.5)	70(58.3)	17(14.2)		
Residence	Urban	35(39.3)	44(49.4)	10(11.3)	11.184	0.004
	Rural	15(16.9)	59(66.2)	15(16.9)		
Type/Sputum smear	Positive	36(49.3)	25(34.2)	12(16.5)	39.140	<0.001
	Negative	9(9.8)	70(76.1)	13(14.1)		
	EPTB	5(38.5)	8(61.5)	0		
Category of TB	New	34(26.8)	74(58.2)	19(15.0)	0.551	0.759
	Retreatment	16(31.4)	29(56.9)	6(11.7)		
Smoking	No	23(28.0)	47(57.3)	12(14.7)	0.046	0.977
	Yes	27(28.1)	56(58.3)	13(13.6)		
History of DM	< 10 years	19(26.8)	38(53.5)	14(19.7)	7.463	0.113
	≥10 years	18(37.5)	24(50.0)	6(12.5)		
	undiagnosed	13(22.0)	41(69.5)	5(8.5)		
HbA1c	<7.0%	10(29.4)	21(61.8)	3(8.8)	1.100	0.902
	7.0–9.9%	18(26.5)	40(58.8)	10(14.7)		
	≥10%	22(28.9)	42(55.3)	12(15.8)		

TB = tuberculosis; DM = diabetes mellitus

**Table 2:** A post-test: Comparison of the number of patients living in different communities in relation to different economic areas.

Comparison group	χ2	P value
Well-developed VS better-off area	10.032	0.002
Well-developed VS poverty area	6.250	0.012
Better-off area VS poverty area	0.061	0.805

**Table 3:** A post-test: Comparison of the number of patients with different types/smear results in relation to different economic areas.

Comparison group	χ2	P value
Well-developed VS better-off area	36.885	<0.001
Well-developed VS poverty area	9.728	0.006
Better-off area VS poverty area	5.860	0.044

Amongst all the patients, 149 (84%) had vitamin D deficiency—91 (51%) with vitamin D deficiency (10–19.9 ng/ml) and 58 (33%) with severe deficiency (< 10 ng/ml). Serum Vitamin D [25-(OH)D_3_] levels and proportions of patients with various grades of vitamin D deficiency in relation to where they lived in terms of economically developed / poor areas are shown in **Table 4.** The median level (and IQR) of serum vitamin D among the 3 different economic areas was similar, but there were significantly higher proportions of patients with vitamin D deficiency or severe deficiency in the poverty area. Of the TB patients, there were 59 who were newly diagnosed with DM at the time of TB registration and their characteristics and vitamin D status are shown in **Table 5.** They were predominantly male, aged 50 years and above and more resided in rural areas, and there was a high degree of deficiency or severe deficiency of vitamin D.

**Table 4:** Vitamin D status in TB patients with DM, stratified by their residence in different economic areas in China.

Vitamin D status	Patients in well- developed area N = 50	Patients in better-off area N = 103	Patients in poverty area N = 25	P value
Median level (ng/ml) (IQR)	11.5 (7.08, 15.65)	12.2 (8.80, 17.80)	11.5 (9.95, 14.55)	0.387
No (%) with normal level (≥30ng/ml)	2 (4.0)	3 (2.9)	0 (0)	0.655
No (%) with insufficiency (20–29.9ng/ml)	6 (12.0)	17 (16.5)	1 (4.0)	<0.001
No (%) with deficiency (10–19.9ng/ml)	22 (44.0)	51 (49.5)	18 (72.0)	<0.001
No (%) with severe deficiency (<10ng/ml)	20 (40.0)	32 (31.1)	6 (24.0)	<0.001

Vitamin D status determined by measurements of 25-(OH)D_3_. DM = Diabetes mellitus; TB = Tuberculosis; IQR = interquartile range

**Table 5:** Characteristics of TB patients newly diagnosed with DM at the time of TB registration in China.

Characteristics/Categories		Number (%) of distributions
Gender	Male	40 (67.8)
	Female	19 (32.2)
Residence	Urban	24 (40.7)
	Rural	35 (59.3)
Age	<50 years	16 (27.1)
	≥50 years	43 (72.9)
Smoking	Yes	30 (50.9)
	No	29 (49.1)
Serum vitamin D level	Normal ≥30ng/ml	3 (5.1)
	Insufficiency 20–29.9ng/ml	5 (8.5)
	Deficiency 10–19.9ng/ml	30 (50.8)
	Severe deficiency <10ng/ml	21 (35.6)

Baseline characteristics of TB patients with DM in relation to being vitamin D deficient (25-(OH)D_3_ <20ng/ml) are shown in **Table 6**. The adjusted odds of a TB patient with DM having vitamin D deficiency were significantly higher in those with a longer history of DM and in those with uncontrolled DM whose HbA1c ≥10%. Other baseline characteristics such as type and category of TB, seasons of TB notification, DM treatment, FBG levels and smoking status were not associated with vitamin D status.

**Table 6:** Baseline characteristics in TB patients with DM in relation to vitamin D deficiency (<20ng/ml).

Characteristics		Total (N = 178)	No. (%) with vitamin D deficiency (N = 149)	Univariate OR (95% CI)	Multivariate OR (95% CI)	P value
Gender	Male	129	108 (83.7)	Reference		
	Female	49	41 (83.7)	1.00 (0.41–2.43)		
Age	<50 years	58	48 (82.8)	Reference		
	≥50 years	120	101 (84.2)	1.11 (0.48–2.56)		
Residence	Urban	89	76 (85.4)	Reference		
	Rural	89	73 (82.0)	0.78 (0.35–1.74)		
Type/sputum smear	Negative	92	77 (83.7)	Reference		
	Positive	73	61 (83.6)	0.99 (0.43–2.27)		
	EPTB	13	11 (84.6)	1.07 (0.22–5.33)		
Category of TB	New	127	107 (84.3)	Reference		
	Retreatment	51	42 (82.4)	0.87 (0.37–2.07)		
Smoking	No	82	69 (84.1)	Reference		
	Yes	96	80 (83.3)	0.94 (0.42–2.10)		
Months of TB registration	May-Oct.	41	33 (80.5)	Reference		
	Nov.-Apr.	137	116 (84.7)	1.34 (0.54–3.30)		
DM history	<10 years	71	54 (76.1)	Reference		
	≥10 years	48	44 (91.7)	3.46 (1.09–11.04)	3.57 (1.07–11.83)	0.038
	Undiagnosed	59	51 (86.4)	2.01 (0.80–5.05)	2.57 (0.96–6.90)	0.062
DM treatment	Yes	117	97 (82.9)	Reference		
	No	61	52 (85.2)	1.19 (0.51–2.80)		
FBG control	≤7.0 mmol/L	9	7 (77.8)	Reference		
	7.1–9.9 mmol/L	79	62 (78.5)	1.04 (0.20–5.48)		
	10.0–11.9 mmol/L	33	29 (87.9)	2.07 (0.31–13.68)		
	≥12 mmol/L	57	51 (89.5)	2.43 (0.41–14.47)		
HbA1c level	<7.0%	34	25 (73.5)	Reference		
	7.0–9.9%	68	52 (76.5)	1.17 (0.45–3.01)	1.23 (0.45–3.36)	0.683
	≥10.0%	76	72 (94.5)	6.48 (1.83–22.91)	7.31 (1.97–27.18)	0.003

TB = tuberculosis; EPTB = extra-pulmonary tuberculosis; DM = Diabetes mellitus; OR = odds ratio; CI = confidence interval

## Discussion

This study aimed to obtain a better understanding of vitamin D status amongst TB patients with DM in the routine programme setting in China based on their residence according to economic development and baseline characteristics. There were some interesting findings.

First, there was a low median level of vitamin D in patients with dual disease, which was lower than levels found previously in non-DM patients with TB and in the general population [14,19]. This finding is also in line with previous research findings [10,12,13]. Both DM and TB are diseases that impact on nutritional intake and both may contribute to a lower serum vitamin D level [5,6]. A recent meta-analysis also confirmed a significantly lower serum vitamin D level in TB patients versus normal controls, but pointed out a weak association of this trend for Asian populations [12]. However, this was not observed in our study, and possible reasons include the poor population and accumulated risk of TB and DM [20]. A study in India found that no major differences in mean vitamin D levels between TB patients with DM and without DM, but the proportion of those with severe vitamin D deficiency was higher in patients with both TB and DM [21].

Second, about one third of all the patients regardless of their residential areas had severe vitamin D deficiency (<10ng/ml), and this in its own right requires urgent attention. Previous research has indicated that rifampicin causes an accelerated loss of vitamin D due to increased body clearance and limited formation of the active form of vitamin D [25-(OH)D_3_ ] [22]. Isoniazid can also cause impairment of 25-hydroxylation leading to impaired immunological function [23]. Serum vitamin D levels might also be further depressed due to enhanced negative impacts when the drugs are used in combination. Naik et al observed that the mean vitamin D level decreased 16% from the time of TB diagnosis to completion of anti-TB treatment six months later [24]. These findings suggest that there may be a need for vitamin D supplementation in those found with severe vitamin D deficiency at the time of TB registration [25,26]. However, further research is required in this area, as there are conflicting reports about this aspect [11,27,28].

Third, a literature review has shown that there are low serum vitamin D levels among persons living in poor areas compared with developed areas [29], similar to our findings in this study. In the poverty county defined by the China State Council, there was a significantly higher proportion of DM-TB patients with vitamin D deficiency compared with patients in the other economic areas. China is a country with high incidence of TB and high prevalence of latent TB infection [30]. A recent meta-analysis confirmed that vitamin D deficiency is strongly associated with an increased risk of progression from latent TB infection to active TB disease with or without household contacts [12]. This raises the question about whether there is a need for early interventions focused on improving nutritional intake and food fortification with vitamin D supplements and requires discussion amongst policy makers.

Fourth, we did not find associations between vitamin D deficiency and baseline characteristics such as age, gender, residential community and number of daily cigarettes smoked, findings also in line with previous research [14]. Contrary to previous research [14,28], we found no strong association in this study with season, possibly because most of our patients lived in urban areas which may not have been so affected by reduced nutritional intake that occurs in the winter time. In the previous report, 32.5% of the study population resided in urban areas, but the figure was 50.0% in the current study. Another intriguing finding that also requires further research was that patients with smear-positive pulmonary TB were not at increased risk of vitamin D deficiency compared with those who had smear-negative disease. This appears to be counter-intuitive as smear-positive TB tends to be associated with increased disease severity or long-term illness [31], which in turn might be expected to increase the risk of vitamin D deficiency.

Having a history of DM for 10 years or more or a serum glycosylated haemoglobin (HbA1c) level ≥10% were independent risk factors for vitamin D deficiency. Reasons may be as follows. Both DM and TB are associated with malnutrition and this association may be stronger with a longer duration of DM and multiple episodes of TB [32]. Amongst our study group, the proportion with older age and retreatment TB at nearly 30% was higher than found in a previous study of TB patients [14], a finding similar to that observed in Guangzhou [33].

HbA1c reflects average blood glucose levels over a period of 2–3 months and may be less affected by infection related hyperglycaemia [34]. HbA1c ≥10% represents poor glycaemic control and is an indicator for administering insulin treatment in DM patients with TB as recommended by the Union and World Diabetes Foundation. A recent systematic review highlighted that HbA1c is a reliable risk factor of all-cause mortality and cardiovascular mortality in both DM and non-DM populations [35]. In patients with DM, the risk of all-cause mortality increased when HbA1c levels were above 8.0% and were highest when levels were above 9.0% [35]. Our findings and the review suggest that a target value of HbA1c for TB patients with DM should be set at <8.0% and clinicians need to be made aware of this fact. Unexpectedly, not being on DM treatment was not associated with vitamin D deficiency. This finding requires further study, including duration and severity of their diseases.

The strengths of this study were multi-center participation and the large number of TB patients with DM consecutively enrolled from routine programme settings to avoid selection bias. Vitamin D status was assessed according to their different economic development areas. To our knowledge, this is the first report on vitamin D status in DM-TB patients from the routine programme setting stratified by economic areas in China. There were, however, some limitations. First, for those patients newly diagnosed with DM, the diagnosis was based on FBG≥7.0 mmol/L at the time of TB notification without validation by other tests such as the oral glucose tolerance test or HbA1c. Previous research has confirmed the feasibility of taking blood samples for FBG at the time of TB diagnosis for the majority of TB patients [36], but the use of FBG alone to diagnose DM may underestimate the prevalence of DM by as much as 50% [37]. Second, we took blood samples for measurement of HbA1c immediately after the diagnosis of TB without systematically performing a physical examination. We may therefore have overlooked some conditions that influence the HbA1c level, such as iron deficiency with or without anaemia [38]. Third, we did not collect data on history of symptoms or signs of TB, a factor which might have impacted on vitamin D levels, nor did we analyse patient’s individual food intake in relation to their vitamin D levels. Fourth, the comparison with some conditions in other published studies may not be a true comparison due to the lack of a control group. In addition, 25-(OH)D_3_ was measured with the ECLIA method only in a COBASE, and results were not confirmed with mass spectrometry, which is more accurate than the ECLIA method, due to this being unavailable in hospital settings in China.

## Conclusions

Our study found that 84% of TB patients with DM had vitamin D deficiency or severe deficiency, with the proportions being higher in patients residing in a designated poverty area compared with other areas. Those with a longer duration of DM and those showing HbA1c level ≥10% were at higher risk of vitamin D deficiency. TB Programme managers and clinicians need to pay more attention to vitamin D status in their patients, especially for those in living in poverty areas.

## Supporting information

S1 File Dataset. (XLS)
